# The Structural Characterization of a Polysaccharide from the Dried Root of *Salvia miltiorrhiza* and Its Use as a Vaccine Adjuvant to Induce Humoral and Cellular Immune Responses

**DOI:** 10.3390/ijms25147765

**Published:** 2024-07-16

**Authors:** Yixuan Zhu, Xiaochen Yang, Pengfei Gu, Xiao Wang, Yongzhan Bao, Wanyu Shi

**Affiliations:** College of Traditional Chinese Veterinary Medicine, Hebei Agricultural University, No. 2596 Lekai South Street, Baoding 071000, China; 20221200088@pgs.hebau.edu.cn (Y.Z.); 20232200613@pgs.hebau.edu.cn (X.Y.); pfgu@hebau.edu.cn (P.G.); wxwangxiao418@163.com (X.W.); baoyongzhan2006@126.com (Y.B.)

**Keywords:** *Salvia miltiorrhiza* polysaccharide, OVA, adjuvant, humoral immune response, cellular immune response

## Abstract

In order to supplement the research gap concerning *Salvia miltiorrhiza* polysaccharide extracted from Danshen in NMR analysis, and to clarify its immune enhancement effect as an adjuvant, we isolated and purified SMPD–2, which is composed of nine monosaccharides such as Ara, Gal, and Glc from Danshen. Its weight average molecular weight was 37.30 ± 0.096 KDa. The main chain was mainly composed of →4)-α-D-Galp-(1→, →3,6)-β-D-Glcp-(1→ and a small amount of α-L-Araf-(1→. After the subcutaneous injection of SMPD–2 as an adjuvant to OVA in mice, we found that it enhanced the immune response by activating DCs from lymph nodes, increasing OVA-specific antibody secretion, stimulating spleen lymphocyte activation, and showing good biosafety. In conclusion, SMPD–2 could be a promising candidate for an adjuvant.

## 1. Introduction

Danshen is the dried root of the Chinese medicinal plant *Salvia miltiorrhiza*; in the clinical practice of traditional Chinese medicine, Danshen has long been used for the prevention and treatment of immune diseases, kidney diseases, etc. [1]. Herbs usually contain a variety of bioactive components with specific biological activities [2]. The common active components of Danshen are polysaccharides, Tanshinones, and phenolic acids [3]. Compared with other Chinese herbal polysaccharides, the study of *Salvia miltiorrhiza* polysaccharides (SMPs) is relatively new [4,5]. The current study found that SMPs are mainly composed of Man, Gal, Ara, Xyl, etc.; this composition changes with different materials and separation methods. The weight average molecular weight of SMPs was in the range of 2~1300 kDa. For the detailed structure of SMPs, only one article reported the NMR results of SMAP-1 extracted from the aerial parts of *Salvia miltiorrhiza* [6], while the NMR structure of SMPs extracted from dry roots was not reported. In addition, SMPs have also been reported to have good anti-tumor effects [7,8,9]; this provides us with a clue that SMPs have immunostimulatory effects and we can further explore their adjuvant potential. At present, adjuvants have become one of the indispensable ingredients in vaccines to induce a stronger, faster, and longer-lasting immune response [10,11]. Traditional adjuvants such as aluminum adjuvant and Freund’s adjuvant still have some defects while inducing strong immune responses [12,13], such as only inducing a single humoral immune response, causing redness and swelling at the injection site, and causing fever [14,15]. Therefore, a new adjuvant with a stronger immunogenicity and safety performance needs to be developed.

In our experiment, we not only used HPACE, SEC-MALLS-RI, GC-MS, etc., to characterize SMPs, but we also carried out a detailed NMR analysis of SMPs and studied the immune enhancement effect of SMPs as a model antigen OVA adjuvant in order to provide a reference for the development of new adjuvants.

## 2. Results

### 2.1. Characterization of SMPs

#### 2.1.1. Purification and Morphology

**Figure 1:**
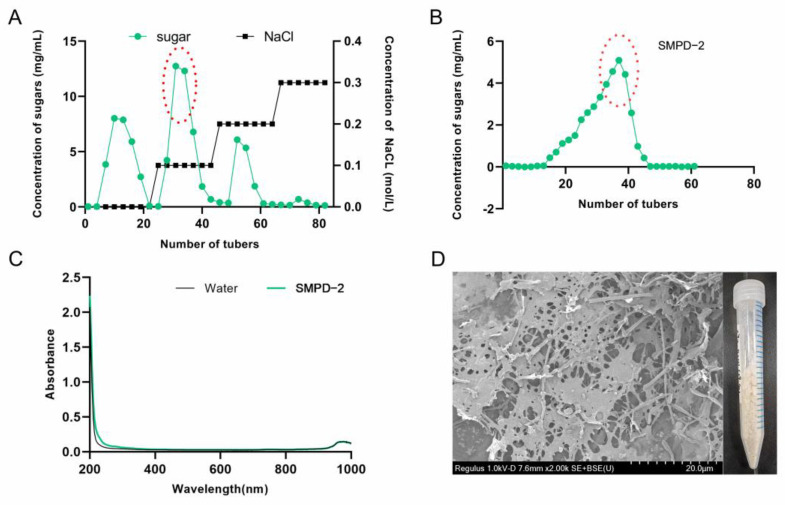
(**A**) Elution curve of SMPs on a DEAE-cellulose column. (**B**) Elution curve of SMPD–2 on a Sephacryl S-400 HR column. (**C**) UV of SMPD–2. (**D**) SEM and the macroscopic state of SMPD–2.

The crude polysaccharide extracted via water extraction and alcohol precipitation was named SMPD. The yield of SMPD was 16.7% and the total sugar content was 89.1%. Then, three active components were obtained after ion purification, named SMPD-1/SMPD–2/SMPD-3, respectively (Figure 1A). We selected SMPD–2, the component with the highest sugar content for gel purification, a yield of 38.4%, and a total sugar content of 93.3% (Figure 1B).

The UV full-wavelength scanning results of SMPD–2 in the figure have no absorption peaks, indicating that it does not contain impurities (Figure 1C). A naked-eye observation showed that SMPD–2 was a pale yellow powder; scanning electron microscopy showed that there were large holes on the surface of the SMPD–2 at a magnification of 2K, and the structure was loose (Figure 1D).

#### 2.1.2. Determination of Monosaccharide Composition and Weight Average Molecular Weight

**Figure 2:**
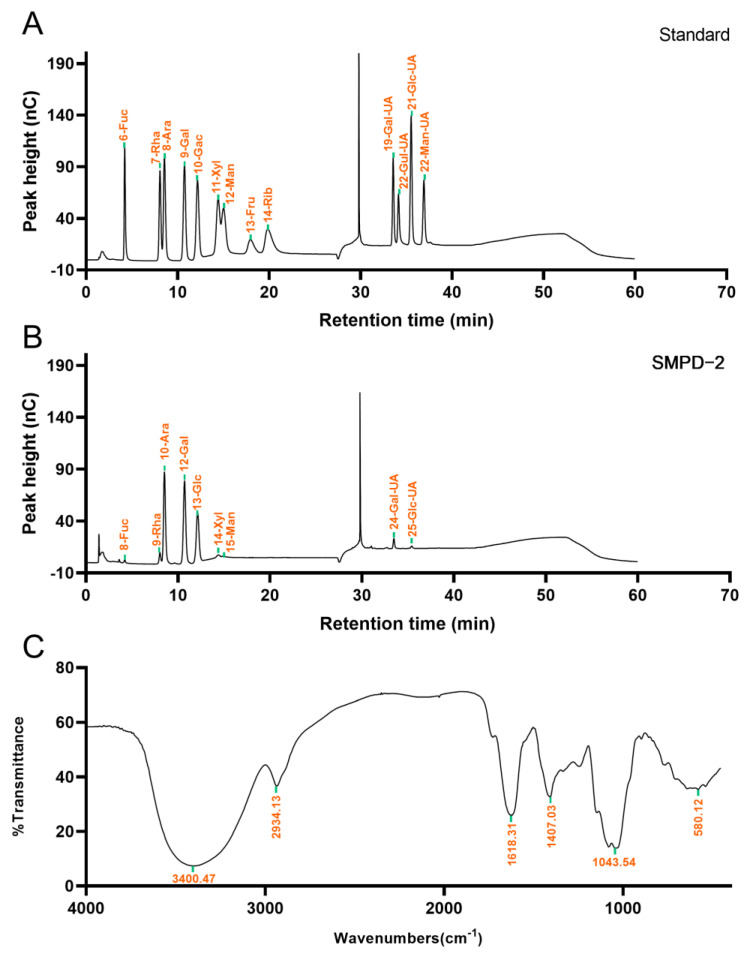
(**A**) Ion chromatogram of standard. (**B**) Ion chromatograms of SMPD–2. (**C**) FT-IR of SMPD–2.

The IC analysis showed that SMPD–2 was composed of Fuc, Rha, Ara, Gal, Glc, Xyl, Man, Gal-UA, Glc-UA, and the molar ratio was 1.10: 4.37: 33.92: 29.46: 20.79: 2.56: 1.76: 5.27: 0.77. Among them, the cumulative molar percentage of Ara, Gal, and Glc reached 83.62% (Figure 2B). In contrast, the molar percentage of others was relatively small, and these components may vary greatly in different determinations. The Mw of SMPD–2 was 37.30 ± 0.096 KDa.

#### 2.1.3. FT-IR Analysis

The functional groups of SMPD–2 were characterized using the FT-IR in the range of 4000 cm^−1^~450 cm^−1^. As shown in Figure 2C, the O-H stretching vibration band of SMPD–2 was at 3300 cm^−1^~3500 cm^−1^. The C-H stretching vibration is located at 2934.13 cm^−1^. The C=O stretching vibration is located at 1618.31 cm^−1^. The tensile vibration of C–O is located at 1043.54 cm^−1^ [16]. 

#### 2.1.4. Glycosidic Linkage Pattern of SMPD–2

The glycosidic linkage pattern of SMPD–2 is shown in Table 1. GC-MS was used for detections within SMPD–2. The main structure of the terminal sugar was t-Ara (f) (25.78%), the main composition of the sugar chain was 4-Gal(p) (25.71%), and the main composition of the branched chain was 3,6-Glc(p) (14.92%). The total ion chromatogram and tandem mass spectra of the characteristic peaks of SMPD–2 are shown in the Supplementary Materials (Figure S1).

#### 2.1.5. NMR Elucidation of SMPD–2

**Figure 3:**
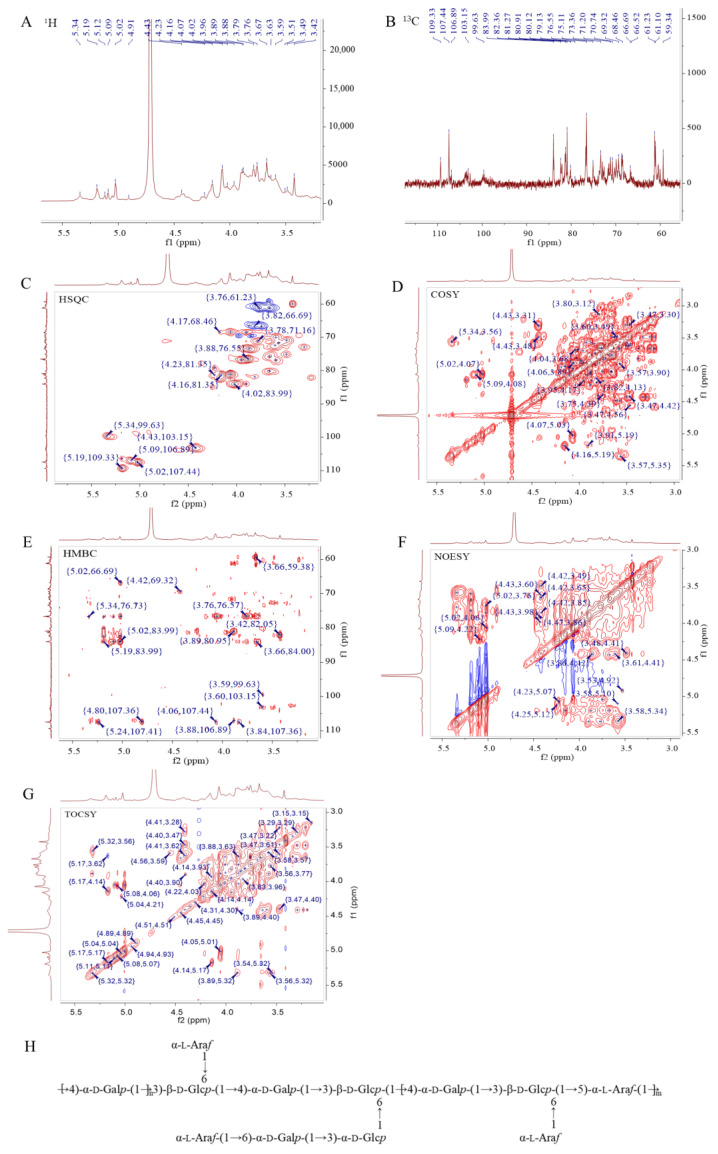
The ^1^H, ^13^C, HSQC, COSY, HMBC, NOESY, and TOCSY analyses of SMPD–2. (**A**) ^1^H of SMPD–2; (**B**) ^13^C of SMPD–2; (**C**) HSQC of SMPD–2; (**D**) COSY of SMPD–2; (**E**) HMBC of SMPD–2; (**F**) NOESY of SMPD–2; (**G**) TOCSY of SMPD–2; (**H**) speculated structure of SMPD–2. The high-definition figures are shown in Figure S2 of the Supplementary Materials.

To speculate the structural characteristics of SMPD–2, 1D and 2D-NMR were performed. The hydrogen spectrum of SMPD–2 identified multiple coupled signal peaks in the anomeric signal region of δ 4.3~5.4 ppm, indicating that SMPD–2 contains a variety of sugar residues (Figure 3A). It was preliminarily judged that there were β-glycosidic bond conformation and α-glycosidic bond conformation in SMPD–2. The non-isomer hydrogen signals were mainly concentrated in the δ 3.1~4.2 ppm region, individual signals overlap with each other seriously, and the H2~H6 chemical shifts of each sugar residue should be attributed by combining COSY, TOCSY, and HSQC, respectively. Then, we combined the ^13^C NMR and HSQC of the hetero head region of the cross peak to determine the hetero head signals of SMPD–2 were δ 5.02/107.44, δ 5.19/109.33, δ 4.43/103.15, δ 5.09/106.89, δ 5.34/99.63, and δ 5.19/106.41 ppm (Figure 3B,C), respectively, recorded as sugar residues A, B, C, D, E, and F. 

##### α-L-Araf-(1→ (Residue A)

The abnormal signal of sugar residue A was δ5.02/107.44 ppm (H1/C1), suggesting that it may be an α-configuration arabinose residue. Combined with the chemical shift in sugar residue A-H1 and the cross peak of COSY, the chemical shifts in H2~H5 were determined to be 4.07 ppm, δ 3.88 ppm, δ 4.03 ppm, δ 3.76/3.66 ppm, respectively. Then, the chemical shifts in C2~C5 on the sugar ring were determined to be δ 80.91 ppm, δ 76.55 ppm, δ 83.99 ppm, and δ 61.23 ppm using HSQC. Among them, the chemical shift in C1 was transferred to the low field, suggesting that the residue was replaced at the O-1 position of the sugar ring. By combining methylation and previous studies [17], it was inferred that the sugar residue A may be α-L-Araf-(1→.

##### →4)-α-D-Galp-(1→ (Residue B)

The anomeric signal of sugar residue B is δ5.19/109.33 ppm (H1/C1), suggesting that it may be an α-configuration galactose residue. By combining the chemical shift in sugar residue B-H1 with the cross peak of COSY, and then analyzing the results of HSQC, we obtained the H2~H6 signal and C2~C6 chemical shift in sugar residue B (Table 2). Among them, the chemical shifts in C1 and C4 were transferred to the low field, suggesting that the residues were replaced at the O-1 and O-4 positions of the sugar ring. By combining methylation and previous studies [18], it was inferred that the sugar residue B may be →4)-α-D-Galp-(1→.

##### →3,6)-β-D-Glcp-(1→ (Residue C)

The anomeric signal of sugar residue C was δ4.43/103.15 ppm (H1/C1), suggesting that residue C may be a β-configuration glucose residue. According to the above method, we obtained that the chemical shifts in the sugar residue C moved to the low field were C1 and C3, indicating that the residues were replaced at the O-1 and O-3 positions of the sugar ring. By combining methylation and previous studies [19], it was inferred that the sugar residue C may be →3,6)-β-D-Glcp-(1→.

According to the above similar steps, it was speculated that the residue D was →5)-α-L-Araf-(1→ [20], the residue E was →3)-α-D-Glcp-(1→ [21], and the residue F was →6)-α-D-Galp-(1→ [22]. 

##### The Connection Mode and Sequence of Sugar Residues

Combined with HMBC (Figure 3E), we speculated that the connection mode of each residue in SMPD–2 were A-H1/C-C6 (δ 5.02/66.69 ppm) and A-H1/F-C6 (δ 5.02/66.52 ppm); B-H1/B-C4 (δ 5.19/76.73 ppm) and B-H1/C-C3 (δ 5.19/80.12 ppm); C-H1/D-C5 (δ 4.43/69.32 ppm) and C-C1/B-H4 (δ 103.15/3.59 ppm); D-H1/B-C4 (δ 5.09/76.73 ppm); and F-H1/E-C3 (δ 5.19/83.99 ppm). Further combined with the NOESY (Figure 3F), it was speculated that the connection order of each residue in the polysaccharide were A-H1/C-H6 (δ 5.02/3.82 3.74 ppm) and A-H1/F-H6 (δ 5.02/3.83 3.69 ppm); B-H1/B-H4 (δ 5.19/3.59 ppm) and B-H1/C-H3 (δ 5.19/3.66 ppm); C-H1/B-H4 (δ 4.43/3.59 ppm) and C-H1/D-H5 (δ 4.43/3.98 3.85 ppm); E-H1/C-H6 (δ 5.34/3.82 3.74 ppm); and F-H1/E-H3 (δ 5.19/3.89 ppm).

Therefore, based on NMR information and the methylation analysis, it was inferred that the SMPD–2 was mainly composed of →4)-α-D-Galp-(1→, →3,6)-β-D-Glcp-(1→ and a small amount of →5)-α-L-Araf-(1→, which were connected to form the main chain. The branched chain is mainly composed of α-L-Araf-(1→, →3)-α-D-Glcp-(1→ and →6)-α-D-Galp-(1→, which were connected to each other and then connected to the O-6 position of the sugar residue →3,6)-β-D-Glcp-(1→. α-L-Araf-(1→, linked at the O-6 position of sugar residue →3,6)-β-D-Glc*p*-(1→. Therefore, it was speculated that the possible structure of the SMPD–2 was shown in Figure 3G.

### 2.2. Activation of Dendritic Cells in Lymph Nodes

Preliminary laboratory research has found that SMPD–2 with the concentration of 1000~1.95 μg/mL was non-toxic to mouse peritoneal macrophages in vitro (Figure S3A) and can significantly enhance the cytokine secretion level of IL-1 and IFN-γ in the supernatant of mouse peritoneal macrophage (Figure S3B,C). SMPD–2 has a good stimulating effect on immune cells in vitro, so we further conducted in vivo experimental studies on SMPD–2.

**Figure 4:**
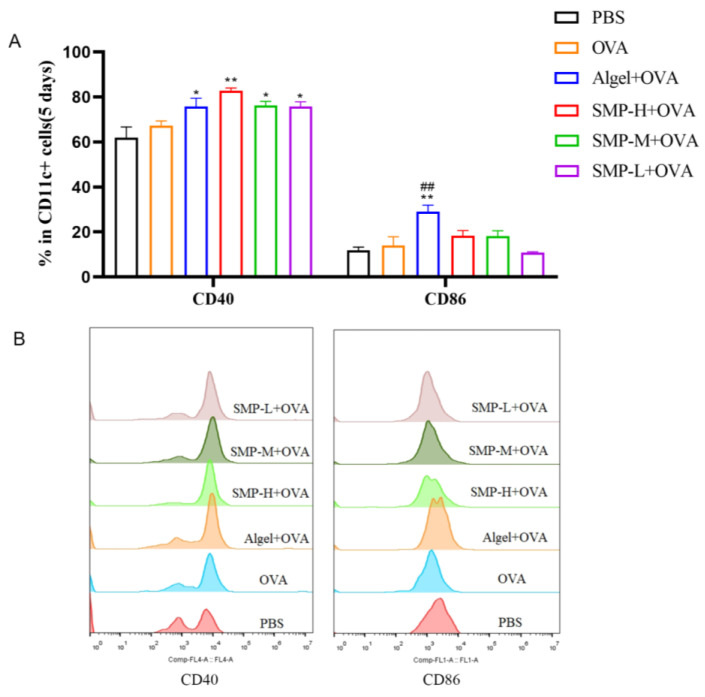
(**A**) The expression of CD40 and CD86. (**B**) The representative FACS plots of different groups. In the figure, SMP-H+OVA represents a high concentration of SMPD–2 mixed with OVA, SMP-M+OVA represents a medium concentration of SMPD–2 mixed with OVA, and SMP-L+OVA represents a low concentration of SMPD–2 mixed with OVA. The data are expressed as the means ± SEM; *n* = 4, * *p* < 0.05, ** *p* < 0.01, compared with the OVA group; ^##^ *p* < 0.01, compared with the SMP-H+OVA group.

On the fifth day after the first immunization, to evaluate the ability of SMPD–2 to activate conventional dendritic cells (DCs) in lymph nodes, the single cell suspension of lymph nodes was collected, washed, stained, and analyzed via flow cytometry. Three concentrations of SMPD–2+OVA and Algel+OVA groups up-regulated the expression of CD40 and CD86, and SMP-H+OVA significantly up-regulated the expression of CD40 (*p* < 0.01); Algel+OVA significantly up-regulated the expression of CD86 (*p* < 0.01) (Figure 4A). 

### 2.3. OVA-Specific IgG and Its Subtypes

**Figure 5:**
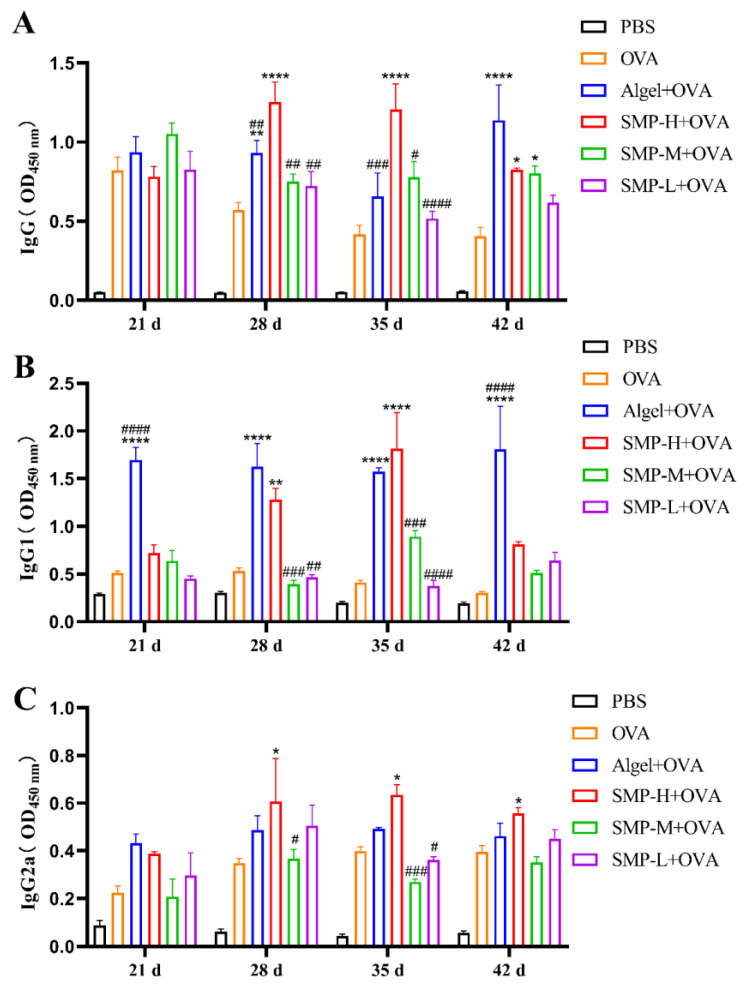
(**A**) OVA-specific IgG titers. (**B**) OVA-specific IgG1 titers. (**C**) OVA-specific IgG2a titers. Data were expressed as the mean ± SEM, *n* = 4, * *p* < 0.05, ** *p* < 0.01 and **** *p* < 0.0001, compared with the OVA group; *^#^ p* < 0.05, ^##^ *p* < 0.01, *^###^ p* < 0.01 and *^####^ p* < 0.0001, compared with the SMP-H+OVA group.

After the immunization, we measured the level of OVA-specific IgG antibodies four times. The level of IgG induced by the SMP-H+OVA immunization group was significantly higher than the OVA, Algel+OVA, SMP-M+OVA, and SMP-L+OVA groups on the 28th and 35th days after the first immunization (*p* < 0.0001). Still, there was a downward trend on the 42nd day (Figure 5A).

The OVA-specific antibody IgG1 (Th2-related subtype) induced by the SMP-H+OVA group showed extremely high antibody titers only on the 28th (*p* < 0.01) and 35th (*p* < 0.0001) days compared with the OVA group. It was higher than the Algel+OVA group on the 35th day (Figure 5B). The antibody titer of specific IgG2a (Th1-related isotype) induced by the SMP-H+OVA group was higher than that of other groups on the 28th to 42nd days (*p* < 0.05) and maintained at a high level (Figure 5C).

### 2.4. Activation of Spleen T Cells after Vaccination

**Figure 6:**
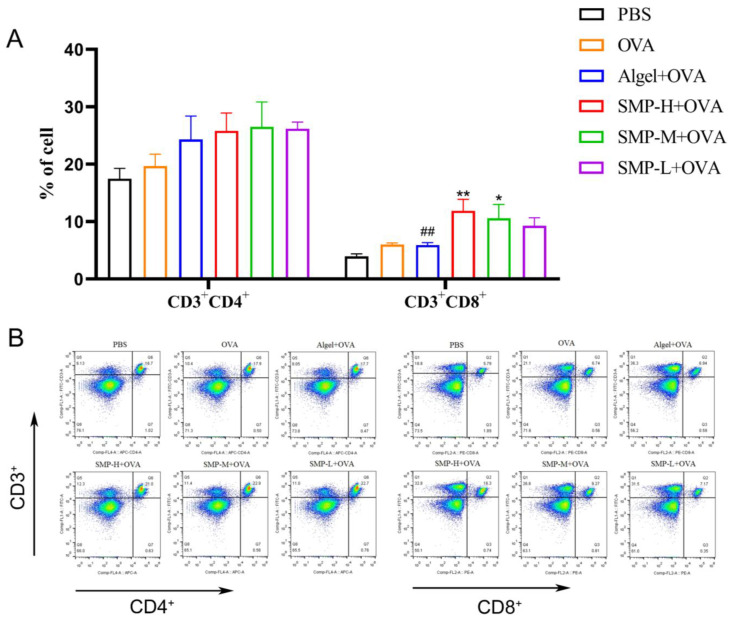
(**A**) Ratio of CD3^+^CD4^+^ to CD3^+^CD8^+^ splenocytes harvested. (**B**) Dot plot analyses of subpopulations of CD4^+^ and CD8^+^ T cells. Data were expressed as the mean ± SEM, *n* = 4, * *p* < 0.05, ** *p* < 0.01, compared with the OVA group; ^##^ *p* < 0.01 compared with the SMP-H+OVA group. The red in the dot plot gradually changes to blue, representing the cell density from dense to sparse.

Mouse spleen lymphocytes were collected on the 28th day after the first immunization and stimulated with OVA for 48 h. CD3^+^CD4^+^ and CD3^+^CD8^+^T cells were determined. As shown in Figure 6A, three concentrations of SMPD–2+OVA and Algel+OVA have a promoting effect on CD3^+^CD4^+^T cells, and SMP-H+OVA can significantly increase CD3^+^CD8^+^T cells compared with the OVA and Algel+OVA groups (*p* < 0.01); SMP-M+OVA can significantly increase CD3^+^CD8^+^T cells compared with OVA (*p* < 0.05). 

### 2.5. Activation of Cytotoxic T lymphocytes

**Figure 7:**
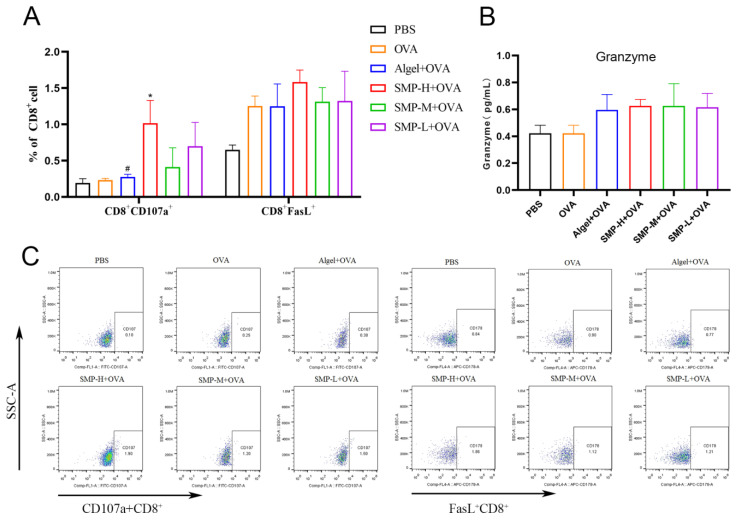
(**A**) Ratio of CD8^+^CD107a^+^ to CD8^+^FasL^+^. (**B**) Concentration of Granzyme in cell supernatant. (**C**) Dot plot analyses of subpopulations of CD107a^+^ and FasL^+^T cells. Data were expressed as the mean ± SEM, *n* = 4, * *p* < 0.05 compared with the OVA group; *^#^ p* < 0.05 compared with the SMP-H+OVA group. The red in the dot plot gradually changes to blue, representing the cell density from dense to sparse.

The expression levels of CD107a^+^ of the SMP-H+OVA group were significantly higher than the OVA and Algel+OVA groups (*p* < 0.05) on the 28th day after the first immunization. The expression levels of FasL^+^ of the SMP-H+OVA group were higher than other groups. SMP-M+OVA and SMP-L+OVA have no significant effect on the expression levels of CD107a^+^ and FasL^+^ in CD8^+^T cells (Figure 7A). Three concentrations of SMPD–2+OVA can all increase the secretion level of Granzyme B (Figure 7B).

### 2.6. Cytokine Levels in Cellular Supernatant

**Figure 8:**
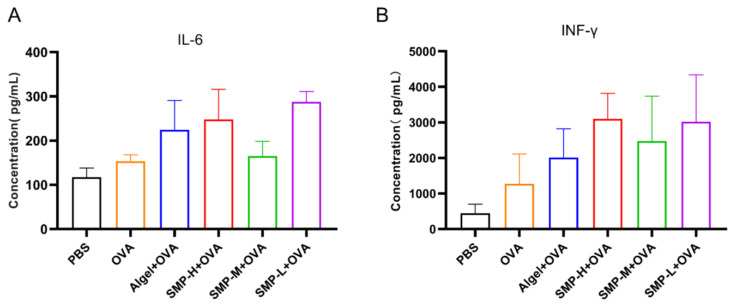
The levels of IL-6 (**A**) and IFN-γ (**B**) in spleen cell supernatant.

On the 28th day after the first immunization, we found that the Algel+OVA, SMP-H+OVA, and SMP-L+OVA groups, compared with the OVA group, could increase the expression levels of IL-6 (Figure 8A) and IFN-γ (Figure 8B) in the cell supernatant.

### 2.7. Biosafety Analysis of SMPD–2

**Figure 9:**
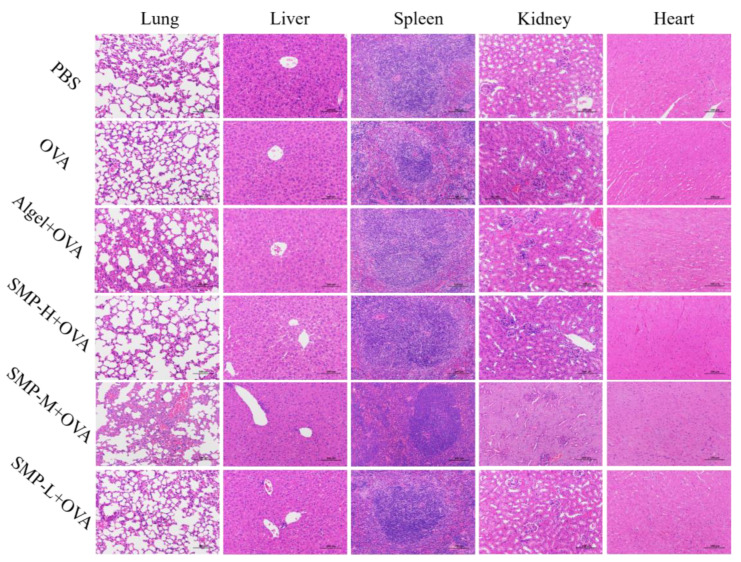
Histopathological analysis. The scale bar was 100.

After immunizing mice with SMPD–2, its degradation products will enter the liver, heart, kidney, and other organs with the migration of APCs and other related pathways. Therefore, it is of great significance to detect the biochemical indices and histological changes in the above organs for the safety evaluation of SMPD–2 in vivo. The results of the biochemical indices showed that there was no significant difference in serum liver function indices (AST, ALT, and ALP), cardiac function indices (LDH), and renal function indices (BUN) between three concentrations of SMPD–2+OVA and PBS (Table 3). Tissue sections showed no cellular damage or inflammation in the liver, heart, lung, kidney, and spleen of mice (Figure 9). Both results proved that SMPD–2 had good biosafety. 

## 3. Discussion

SMPs have been shown to regulate the immune function of higher vertebrates [23], such as increasing the proliferation activity of lymphocytes in cancer patients [24]. However, it is regrettable that few articles have detailed reports on the NMR structure characteristic of SMPs and their effects as adjuvants. So, we have supplemented this part of the research gap.

In this study, a polysaccharide (SMPD–2) was isolated and purified via water extraction and alcohol precipitation. A monosaccharide analysis revealed Ara, Gal, and Glc were the main monosaccharides of SMPD–2, and there was a small amount of Fuc, Rha, Xyl, Man, Gal-UA, and Glc-UA in SMPD–2; the presence of uronic acid suggests that it is an acidic polysaccharide. This result is not exactly similar to previous studies, but more similar to the research of Wang et al. (2020), possibly because we all chose the dried root produced in Shandong Province [25]. Then, we analyzed the glycosidic bonds and linkages of SMPD–2 via GC-MS and NMR. It was found that the main chain was mainly composed of →4)-α-D-Galp-(1→ and that →3,6)-β-D-Glcp-(1→. →4)-α-D-Galp-(1→ is the same as the main chain of GLP-2 extracted from submerged cultured *Ganoderma lucidum* [26] and →3,6)-β-D-Glcp-(1→ is the same as the main chain of RLP50-2 extracted from the fruits of *Rosa laevigata* [27]. It has been reported that both GLP-2 and RLP50-2 have good immunomodulatory effects. Therefore, we speculate that the immune enhancement effect of SMPD–2 as an adjuvant is related to its main chain composition. In addition, the higher branching degree of SMPD–2 makes it have good water solubility and increases the contact surface area to better exert its biological activity [28]. In summary, the high water solubility of SMPD–2 and the linkages of →4)-α-D-Galp-(1→ and →3,6)-β-D-Glcp-(1→ may be the reasons for its adjuvant potential.

Macrophages play an important role in the immune system because of their immunomodulatory regulation [29]. In vitro, we validated that SMPD–2 can enhance the phagocytic activity of macrophages. To further verify the adjuvant potential of SMPD–2, we first evaluated its activation effect on lymph node DCs as an adjuvant to the model antigen OVA [30]. The research results indicate that compared to using OVA alone, adding a high concentration of SMPD–2 can significantly promote the activation of DCs in lymph nodes, thereby inducing a stronger immune response level. 

The expression level of IgG and its subtypes in serum is one of the main criteria for evaluating the effect of the vaccine. From the results, it is not difficult to find that SMPD–2 can increase the antibody titer of IgG and its subtypes, but compared with the 21 d, 28 d, and 35 d, the antibody titer on the 42nd day decreased significantly, which was consistent with other water-soluble polysaccharides. Due to its short half-life in vivo, it cannot induce long-term humoral immune responses [31,32]. After confirming that SMPD–2 can induce humoral immune responses, we further investigated its impact on cellular immune responses in vivo. When CD4^+^T cells are activated in the body, they differentiate into Th1 and Th2 cells and secrete cytokines such as IFN-γ (Th1) and IL-6 (Th2); these have antiviral effects or promote the transformation of innate immune responses into acquired immune responses, respectively [33,34]. We detected that three concentrations of SMPD–2 mixed with OVA can promote the secretion of TNF-γ and IL-6 in the supernatant of spleen cells. After conducting experiments on CD8^+^T cells, we found that the high concentration of SMPD–2 mixed with OVA could better activate the activation of spleen CD8^+^T cells compared with the OVA group. The CD8^+^T cells differentiate into CTLs after the stimulation and kill virus-infected cells through the perforin Granzyme B killing pathway and FasL-mediated target cell apoptosis pathway [35,36], and the activation of CTLs will enhance the expression level of CD107a^+^ in CD8^+^T cells. Therefore, the expression level of CD107a^+^ is the main reference indicator for their activation [37]. We found that three concentrations of SMPD–2 mixed with OVA could up-regulate the expression level of CD107a^+^, FasL^+^, and Granzyme B in CD8^+^T cells compared with the OVA group, and the high-concentration group was the most significant (*p* < 0.05). 

In addition, the difference between this study and other research is that in the spleen test, the low and medium concentrations of SMPD–2 are unstable. The effect of low SMPD–2 concentration on spleen cell activation and cytokine stimulation was sometimes higher than that of the medium-concentration group, which may be related to the non-focused action scope and instability of SMPD–2 [15,32].

## 4. Materials and Methods

### 4.1. Materials

*Salvia miltiorrhiza* was purchased from Yuluotong Biotechnology Co., Ltd. (Anguo, China); petroleum ether, chloroform, n-butanol, sodium chloride, and sodium nitrate were collected from Sinopharm Chemical Reagent Co., Ltd. (Shanghai, China); DMSO, dichloromethane, and ammonia were purchased from Anpu Experimental Technology Co., Ltd. (Shanghai, China); IgG\IgG1\IgG2a antibodies were purchased from ABclonal (Wuhan, China); DMEM/high-glucose medium was purchased from HyClone (Shanghai, China); penicillin–streptomycin solution and 4% paraformaldehyde were purchased from Biosharp Life Sciences (Beijing, China); a Mouse ELISA Kit was purchased from Lianke Biotechnology Co., Ltd. (Hangzhou, China); antibodies for flow cytometry were purchased from Elabscience (Wuhan, China); Algelinium hydroxide gel adjuvant was purchased from InvivoGen (San Diego, CA, USA). Nicolet iZ-10 spectrometer, Thermo (Shanghai, China).

### 4.2. Extraction and Purification of SMPs

The sliced dried root of *Salvia miltiorrhiza* was produced in Anguo, China. We purchased it from Yuluotong Biotechnology Co., Ltd. Firstly, the root powder was soaked in ethanol solution overnight for degreasing and decolorization and then extracted with hot water (85 °C, 2 h) according to the ratio of material to water of 1:10 [38]. After extracting twice, the concentrated supernatant was mixed with anhydrous ethanol at a ratio of 1:4 for alcohol precipitation [39]. Finally, it was deproteinized via the Sevage method 8 times, degreased with petroleum ether reagent, depigmented with AB-8 macroporous resin, dialyzed with water (3000 Da), and the crude polysaccharide was obtained.

The crude polysaccharide was eluted with 4 mL/min of distilled water and 0.1, 0.2, and 0.3 M NaCl in the DEAE-cellulose column (26 mm × 400 mm), respectively. The obtained components were determined using the phenol-sulfuric acid method for carbohydrate content. The component with the highest content was eluted using distilled water at 1.0 mL/min on a polyacrylamide dextran S-400 HR column (26 mm × 1000 mm) and freeze-dried into a homogeneous polysaccharide.

### 4.3. Structural Characterization of SMPs

Then, we tested the characterization structure of the SMPs. First of all, we determined the protein and impurity content of polysaccharides via an ultraviolet absorption method and then observed the surface morphology of SMPs using a scanning electron microscope [40]. We determined the Fourier transform infrared spectrum of polysaccharides with a Nicolet iZ-10 spectrometer.

Secondly, the monosaccharide composition and Mw of SMPs were determined by HPACE and SEC-MALLS-RI, respectively, and then the specific structure of SMPS was deduced by GC-MS and NMR [41,42].

### 4.4. Animal and Vaccinations 

ICR mice (female, 6 weeks old, 20 g) were obtained from SiPeiFu Co., Ltd. (Beijing, China). All experiments involving animals were performed following the National Research Council’s Guide for the Care and Use of Laboratory Animals and approved by the Animal Welfare and Ethics Committee at Hebei Agricultural University (confirmation number: 2022161).

The 120 female ICR mice were randomly divided into 6 groups: high-concentration SMPs + OVA (2 mg of SMPs + 20 μg of OVA in 0.2 mL), medium-concentration SMPs + OVA (1 mg of SMPs + 20 μg of OVA in 0.2 mL), and low-concentration SMPs + OVA (0.5 mg of SMPs + 20 μg of OVA in 0.2 mL). PBS was used as a negative control, and OVA (20 μg of OVA in 0.2 mL) and Algel + OVA (400 μg of Algel + 20 μg of OVA in 0.2 mL) were used as positive controls. Each mouse was subcutaneously injected with 100 μL on the left and right thighs, respectively, and received an equivalent enhanced dose on the 14th day after the first immunization. 

### 4.5. Immunophenotypic Analysis of Lymph Node Cells

On the 5th day after the first immunization, the inguinal lymph nodes and popliteal lymph nodes of mice were collected from each group. After grinding, the cell suspension was washed with PBS. Then, the anti-CD11c-PE-Cynine7, anti-CD40-APC, and anti-CD86-PE antibodies were added; these were stained, washed, fixed with 1% paraformaldehyde, and determined via flow cytometry. 

### 4.6. Determination of Antibody Responses

The serum of each group at 21, 28, 35, and 42 days after the first immunization was incubated in a 96-well plate that adsorbed OVA antigen (1 μg/mL) in advance for 30 min [43]. After washing, goat anti-mouse IgG, IgG1, and IgG2a were added to the plates and incubated for 1 h. TMB was added for color development for 10~25 min. After termination with termination solution, it was determined at OD450 nm.

### 4.7. Splenic Lymphocyte Immunophenotype Analysis

Splenic cell suspensions of vaccinated mice were obtained on the 28th day after the first immunization [39]. Cells were analyzed via flow cytometry using the following two groups of staining methods (Table 4). The collected cell supernatant was used to determine the secretion levels of Granzyme B, IFN-γ, and IL-6.

### 4.8. Biochemistry Index

The serum of the mice in each group on the 42nd day was obtained and a biochemical analyzer was utilized to detect liver function indicators such as AST, ALT, and ALP, as well as heart function indicators (LDH) and kidney function indicators (BUN). All indicators were compared with the PBS group to test the safety of different concentrations of SMPs in mice.

### 4.9. Histological Analysis of Organs

On the 42nd day after the first immunization of mice, the heart, liver, spleen, lungs, and kidneys of the mice in each group were collected and observed under the microscope after HE staining to evaluate the safety of different concentrations of SMPs on tissues.

### 4.10. Statistical Analysis

The data are represented as means ± standard deviations (SD). One-way ANOVA and Tukey’s multiple-comparison test were carried out to determine the differences among various groups. An AP-value of 0.05 was believed to be statistically significant.

## 5. Conclusions

In this study, we isolated and purified a homogeneous water-soluble Salvia miltiorrhiza polysaccharide SMPD–2 and analyzed its basic structure via methylation and NMR. As an adjuvant of OVA, SMPD–2 can promote the activation of DCs in draining lymph nodes and trigger a strong specific humoral immune response. In addition, SMPD–2 adjuvant can also improve the activation of CD4^+^ and CD8^+^ T cells and the CTLs response of CD8^+^ cells. It also shows good biological safety. At this stage of our study, our results provide a clue that SMPD–2 appears to be worthy of consideration as an effective adjuvant to enhance the immune response of antigens.

## Figures and Tables

**Table 1:** Glycosidic linkages among the monomer residues and molar proportions of SMPD–2.

Glycosidic Linkages	Derivative Name	M_W_	mol, (%)
**t-Ara(f)**	**1,4-di-O-acetyl-2,3,5-tri-O-methyl arabinitol**	**279**	**25.78**
t-Xyl(p)	1,5-di-O-acetyl-2,3,4-tri-O-methyl xylitol	279	0.81
t-Glc(p)	1,5-di-O-acetyl-2,3,4,6-tetra-O-methyl glucitol	323	3.53
t-Gal(p)	1,5-di-O-acetyl-2,3,4,6-tetra-O-methyl galactitol	323	4.46
5-Ara(f)	1,4,5-tri-O-acetyl-2,3-di-O-methyl arabinitol	307	8.05
2-Xyl(p)	1,2,5-tri-O-acetyl-3,4-di-O-methyl xylitol	307	0.77
3-Glc(p)	1,3,5-tri-O-acetyl-2,4,6-tri-O-methyl glucitol	351	6.36
**4-Gal(p)**	**1,4,5-tri-O-acetyl-2,3,6-tri-O-methyl galactitol**	**351**	**25.71**
6-Gal(p)	1,5,6-tri-O-acetyl-2,3,4-tri-O-methyl galactitol	351	4.14
2,4-Rha(p)	1,2,4,5-tetra-O-acetyl-6-deoxy-3-O-methyl rhamnitol	349	2.10
3,4-Glc(p)	1,3,4,5-tetra-O-acetyl-2,6-di-O-methyl glucitol	379	1.14
4,6-Glc(p)	1,4,5,6-tetra-O-acetyl-2,3-di-O-methyl glucitol	379	2.24
**3,6-Glc(p)**	**1,3,5,6-tetra-O-acetyl-2,4-di-O-methyl glucitol**	**379**	**14.92**

**Table 2:** Chemical shifts of ^1^H and ^13^C of sugar residues.

Code	Glycosyl Residues	Chemical Shifts (ppm)					
		H1/C1	H2/C2	H3/C3	H4/C4	H5/C5	H6/C6
A α-L-Ara*f*-(1→		5.02/107.44	4.07/80.91	3.88/76.55	4.03/83.99	3.76, 3.66/61.23	n.d/n.d
B →4)-α-D-Gal*p*-(1→		5.19/109.33	4.16/68.46	3.9/73.36	3.59/76.73	3.49/70.74	3.76, 3.62/61.1
C →3,6)-β-D-Glc*p*-(1→		4.43/103.15	3.48/75.11	3.66/80.12	3.78/71.2	3.87/73.36	3.82, 3.74/66.69
D →5)-α-L-Ara*f*-(1→		5.09/106.89	4.08/81.27	4.23/79.23	4.16/81.35	3.98, 3.85/69.32	n.d/n.d
E →3)-α-D-Glc*p*-(1→		5.34/99.63	3.56/71.55	3.89/83.99	3.79/71.2	3.83/73.7	3.67/59.34
F →6)-α-D-Gal*p*-(1→		5.19/106.41	3.91/72.95	3.65/75.81	3.79/71.17	3.9/73.75	3.83, 3.69/66.52

Note: n.d is the abbreviation of ‘not detected’, which means that it was unrecognized.

**Table 3:** Serum biochemical parameters of the immunized mice (*n* = 4).

Group	ALT (U/L)	AST (U/L)	ALP (U/L)	UREA (mmol/L)	LDH (U/L)
PBS	21.15 ± 5.972	67.89 ± 19.002	79.96 ± 7.216	3.40 ± 0.146	499.58 ± 64.260
OVA	26.61 ± 2.725	99.53 ± 1.056	171.24 ± 31.824	3.86 ± 0.528	806.21 ± 174.741
Algel+OVA	29.53 ± 4.626	94.46 ± 12.682	107.13 ± 21.800	3.79 ± 0.474	787.40 ± 56.323
SMP-H+OVA	35.36 ± 2.449	82.72 ± 26.902	80.11 ± 13.021	4.23 ± 0.287	672.34 ± 142.625
SMP-M+OVA	23.00 ± 7.500	100.34 ± 31.49	139.90 ± 34.009	3.30 ± 0.093	647.88 ± 100.273
SMP-L+OVA	27.77 ± 1.134	96.78 ± 53.936	92.37 ± 7.251	3.96 ± 0.243	737.83 ± 351.49

**Table 4:** Grouping of flow cytometry.

Dyeing Group	Target for Testing	Color Matching [44]
1	T lymphocyte	anti-CD3-FITC, anti-CD4-APC, anti-CD8a-PE
2	Cytotoxic T Lymphocyte	anti-CD8a-PE, anti-CD107-FITC, anti-FASL-APC

## Data Availability

Data will be made available on request.

## Supplementary Materials

The following supporting information can be downloaded at: https://www.mdpi.com/article/10.3390/ijms25147765/s1 [42,45,46,47,48].

## Abbreviations

SMPs, *Salvia miltiorrhiza* polysaccharides; OVA, albumin from chicken egg whites; Algel, Algelinium hydroxide gel adjuvant; SEM, transmission electron microscope.
