# Supplementary material for: The Structural Characterization of a Polysaccharide from the Dried Root of Salvia miltiorrhiza and Its Use as a Vaccine Adjuvant to Induce Humoral and Cellular Immune Responses
Source: Int J Mol Sci. 2024 Jul 16;25(14):7765. doi: 10.3390/ijms25147765 (PMC11277338; doi:10.3390/ijms25147765)
Supplement: Supplementary file 1 [file ijms-25-07765-s001.zip › ijms-3068713-supplementary.pdf]

Supplementary data

The Structural Characterization of a Polysaccharide from the Dried Root of *Salvia miltiorrhiza* and Its Use as a Vaccine Adjuvant to Induce Humoral and Cellular Immune Responses

Yixuan Zhu, Xiaochen Yang, Pengfei Gu, Xiao Wang, Yongzhan Bao and Wanyu Shi *

College of Traditional Chinese Veterinary Medicine, Hebei Agricultural University, No. 2596 Lekai South Street, Baoding 071000, China; 20221200088@pgs.hebau.edu.cn (Y.Z.); 20232200613@pgs.hebau.edu.cn (X.Y.); pfgu@hebau.edu.cn (P.G.); wxwangxiao418@163.com (X.W.); baoyongzhan2006@126.com (Y.B.)

* Correspondence: shiwanyu2010@126.com or shiwanyu@hebau.edu.cn; Tel.: +86-312-7528355

1 Methods

1.1 Preparation of SMPD-2 for methylation

The SMPD-2 was dissolved in DMSO, the solution was methylated in DMSO/NaOH with CH3I. After complete methylation, the permethylated products were hydrolyzed with 2 mol/L TFA at 121°C for 1.5 h, reduced by NaBD4 and acetylated with acetic anhydride for 2.5 h (100 °C) [42].

1.2 GC-MS Conditions

The acetates were dissolved in chloroform and analyzed with GC–MS on an Agilent 6890A-5975C equipped with Agilent BPX70 chromatographic column (30 m × 0.25 mm × 0.25 μm, SGE, Australia), and high purity helium (split ratio 10:1) was used as the carrier gas with an injection volume of 1 μL. Mass spectrometry analysis was performed at the initial temperature of 140 °C for 2.0 min, and the temperature is increased to 230 °C by 3 °C/min for 3 min [45]. The scan mode was SCAN with a range

(m/z) from 50 to 350.

1.3 Macrophage toxicity test

Peritoneal macrophages were isolated and purified from ICR mice according to previous reports [46]. SMPs was introduced to the cells at the concentrations of 1000~1.95 μg/mL. PBS-treated cells were used as controls. Cells were incubated for 48 h, and the cell viability was assayed by the MTT method at 570 nm using a microplate reader.

1.4 Determination of cytokine levels

Mouse peritoneal macrophages were co-incubated with SMPs (500 μg/mL, 250 μg/mL, 125 μg/mL), and PBS was used as a blank control. After 48 h of culture, the contents of cytokines IFN-γ and IL-1β in the supernatant of macrophage culture were detected by ELISA kit.

2 Results

2.1 Complementary information on SMPD-2 methylation

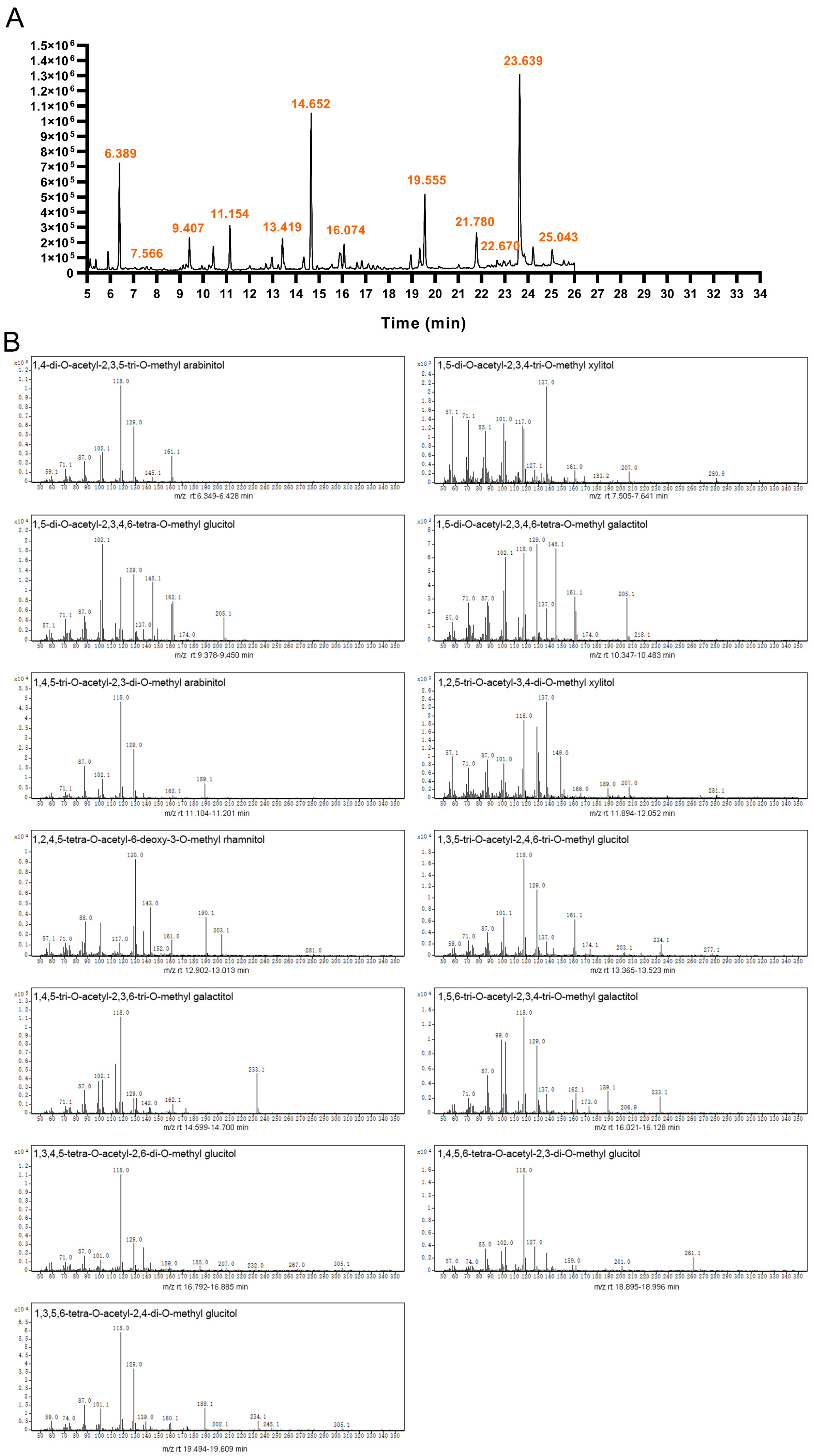

A
6.389
7.566
9.407
11.154
13.419
14.652
16.074
19.555
21.780
22.670
23.639
25.043
Time (min)
B
1,4-di-O-acetyl-2,3,5-tri-O-methyl arabinitol
m/z rt 6.349-6.428 min
1,5-di-O-acetyl-2,3,4-tri-O-methyl xylitol
m/z rt 7.505-7.641 min
1,5-di-O-acetyl-2,3,4,6-tetra-O-methyl glucitol
m/z rt 9.378-9.450 min
1,5-di-O-acetyl-2,3,4,6-tetra-O-methyl galactitol
m/z rt 10.347-10.483 min
1,4,5-tri-O-acetyl-2,3-di-O-methyl arabinitol
m/z rt 11.104-11.201 min
1,2,5-tri-O-acetyl-3,4-di-O-methyl xylitol
m/z rt 11.894-12.052 min
1,2,4,5-tetra-O-acetyl-6-deoxy-3-O-methyl rhamnitol
m/z rt 12.902-13.013 min
1,3,5-tri-O-acetyl-2,4,6-tri-O-methyl glucitol
m/z rt 13.365-13.523 min
1,4,5-tri-O-acetyl-2,3,6-tri-O-methyl galactitol
m/z rt 14.599-14.700 min
1,5,6-tri-O-acetyl-2,3,4-tri-O-methyl galactitol
m/z rt 16.021-16.128 min
1,3,4,5-tetra-O-acetyl-2,6-di-O-methyl glucitol
m/z rt 16.792-16.885 min
1,4,5,6-tetra-O-acetyl-2,3-di-O-methyl glucitol
m/z rt 18.895-18.996 min
1,3,5,6-tetra-O-acetyl-2,4-di-O-methyl glucitol
m/z rt 19.494-19.609 min

Figure S1 (A) Total ion Chromatogram of SMPD-2; (B) Tandem mass spectra of the characteristic peaks of SMPD-2.

2.2 The high-definition figures of NMR

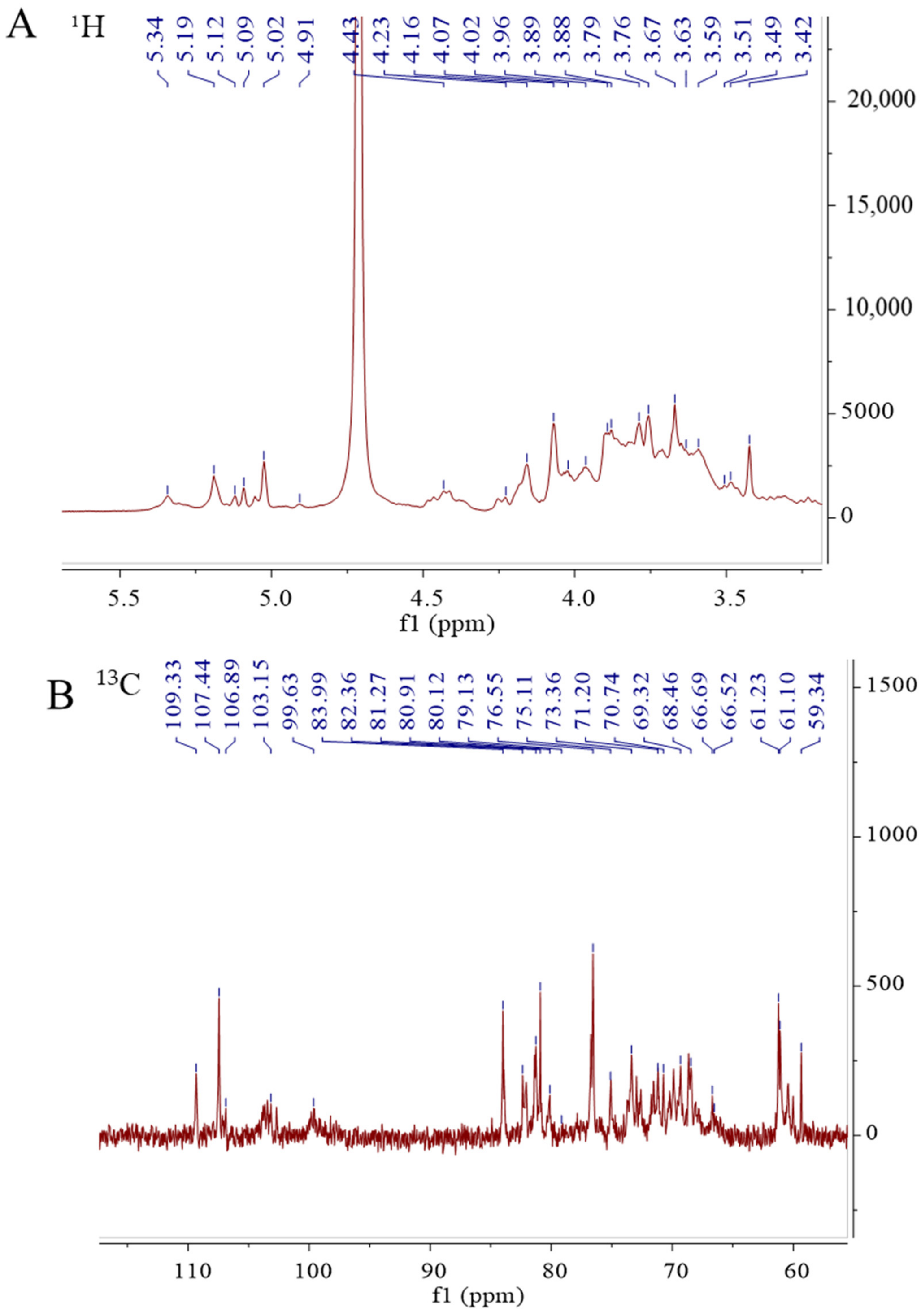

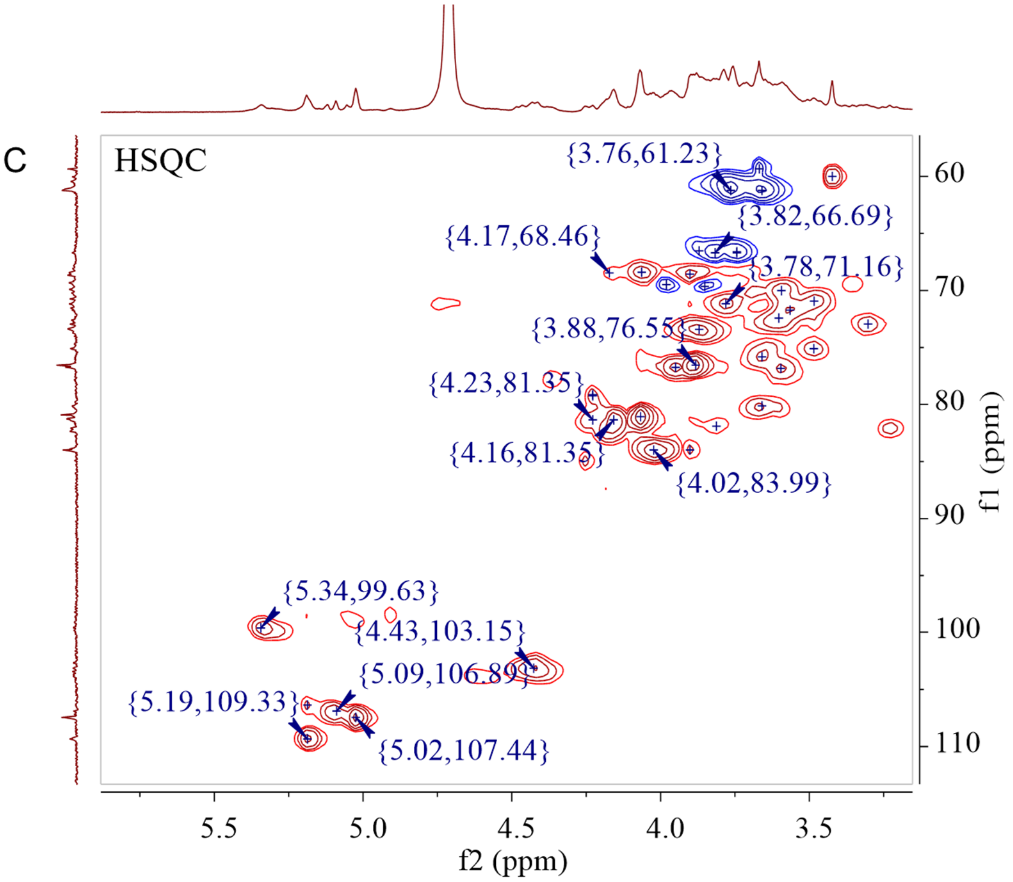
C
HSQC
{3.76,61.23}
{3.82,66.69}
{4.17,68.46}
{3.78,71.16}
{3.88,76.55}
{4.23,81.35}
{4.16,81.35}
{4.02,83.99}
{5.34,99.63}
{4.43,103.15}
{5.09,106.89}
{5.19,109.33}
{5.02,107.44}
f2 (ppm)
f1 (ppm)

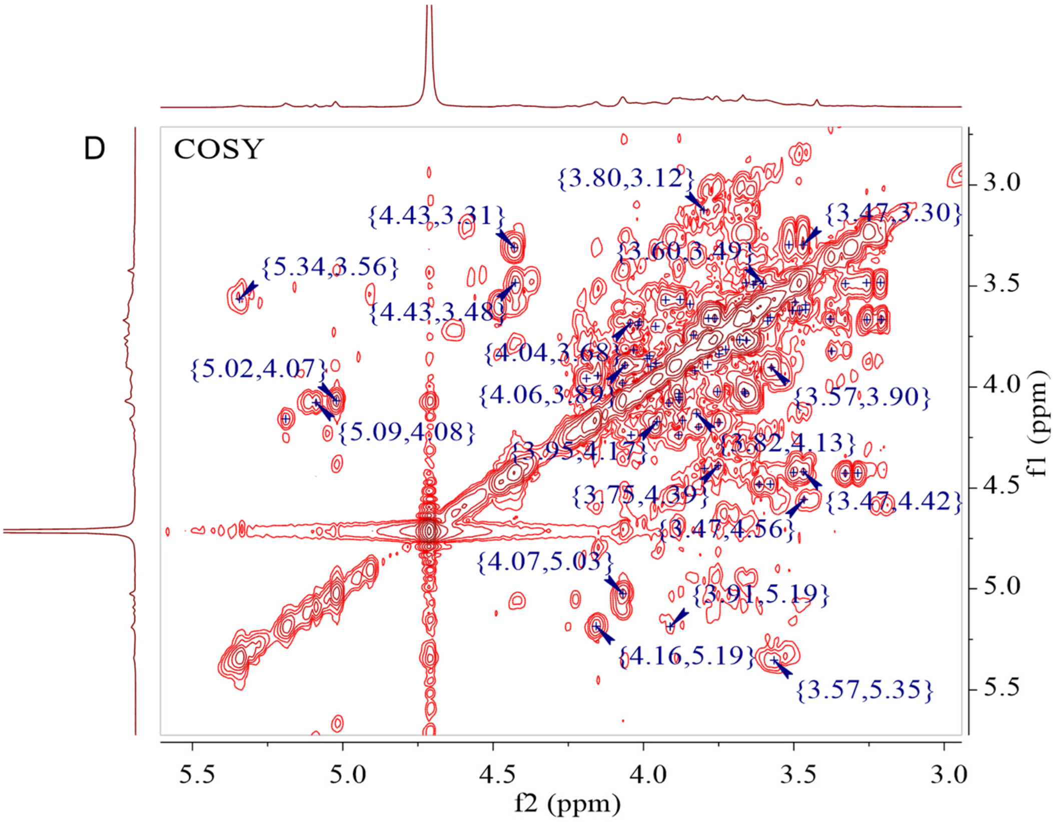
D
COSY
{3.80,3.12}
{4.43,3.31}
{3.47,3.30}
{5.34,3.56}
{3.60,3.49}
{4.43,3.48}
{4.04,3.68}
{5.02,4.07}
{4.06,3.89}
{3.57,3.90}
{5.09,4.08}
{3.95,4.17}
{3.82,4.13}
{3.75,4.39}
{3.47,4.42}
{3.47,4.56}
{4.07,5.03}
{3.91,5.19}
{4.16,5.19}
{3.57,5.35}
f2 (ppm)
f1 (ppm)

E

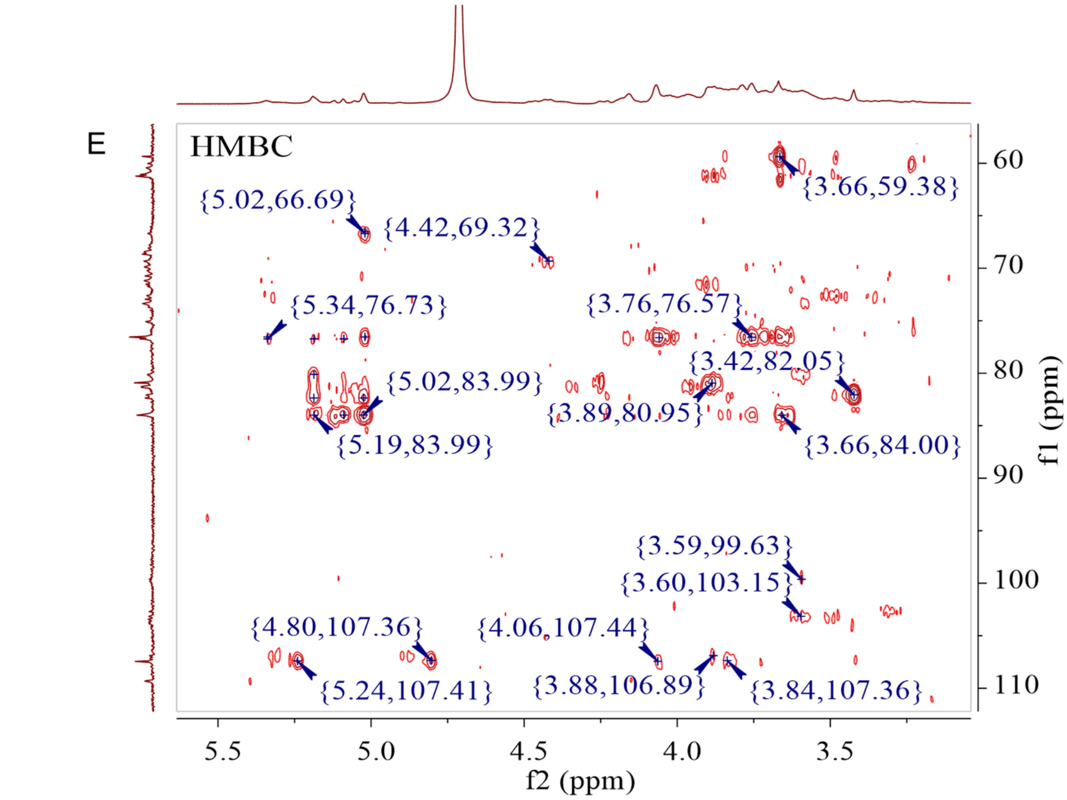
HMBC
{5.02,66.69}
{4.42,69.32}
{3.66,59.38}
{5.34,76.73}
{3.76,76.57}
{3.42,82.05}
{5.02,83.99}
{3.89,80.95}
{5.19,83.99}
{3.66,84.00}
{3.59,99.63}
{3.60,103.15}
{4.80,107.36}
{4.06,107.44}
{5.24,107.41}
{3.88,106.89}
{3.84,107.36}
f2 (ppm)
f1 (ppm)

F

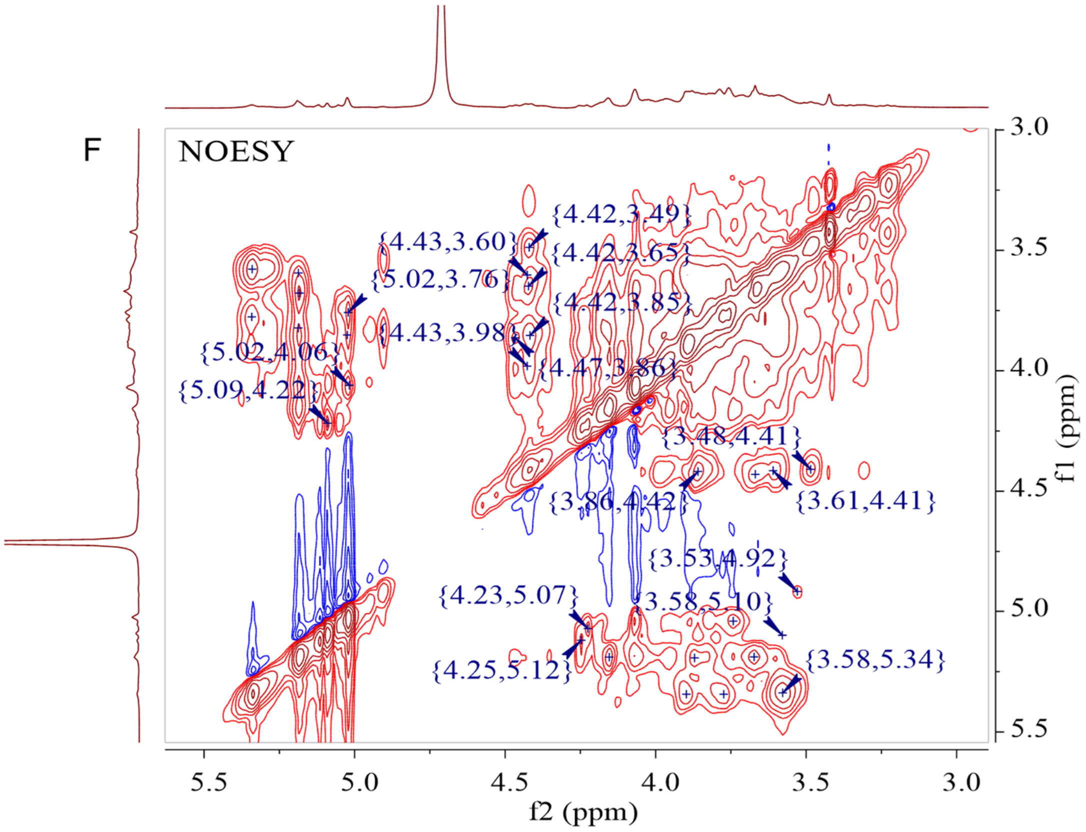
NOESY
{4.42,3.49}
{4.43,3.60}
{4.42,3.65}
{5.02,3.76}
{4.42,3.85}
{4.43,3.98}
{5.02,4.06}
{4.47,3.86}
{5.09,4.22}
{3.48,4.41}
{3.86,4.42}
{3.61,4.41}
{3.53,4.92}
{4.23,5.07}
{3.58,5.10}
{4.25,5.12}
{3.58,5.34}
f2 (ppm)
f1 (ppm)

G

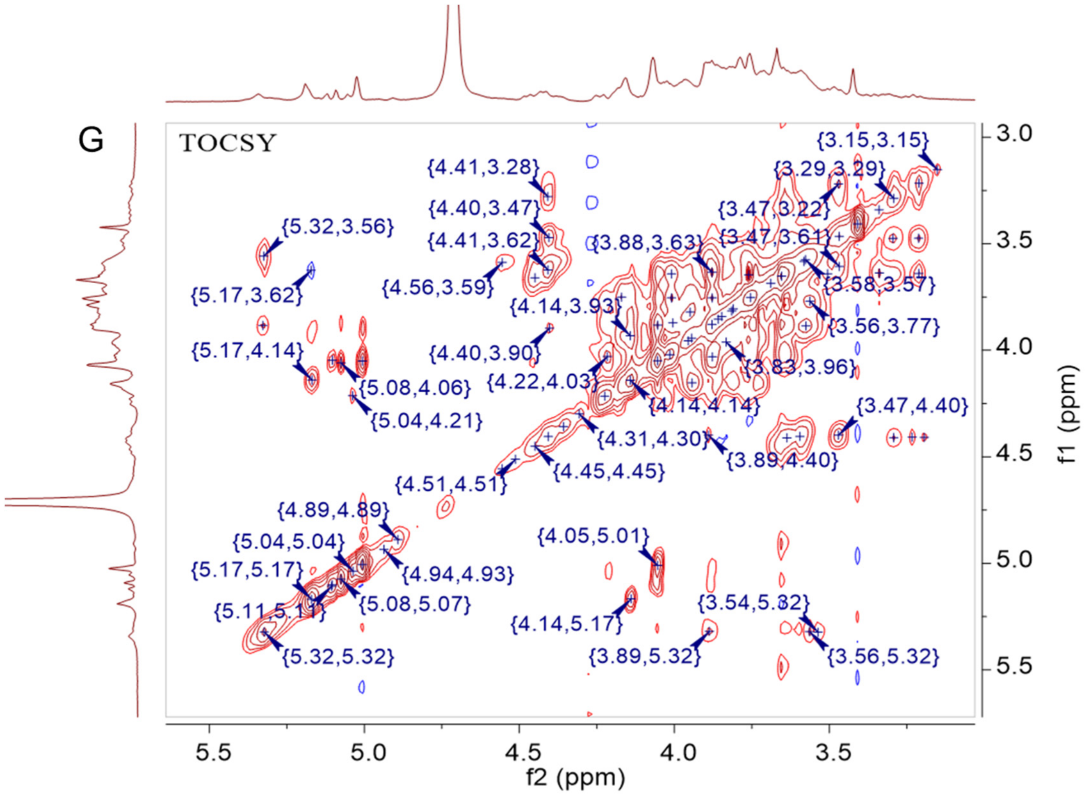

H

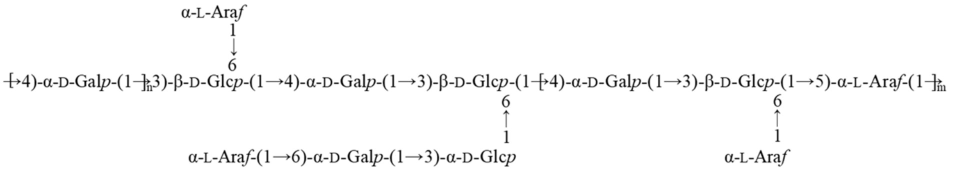

Figure S2 (A) ^{1}H of SMPD-2; (B) ^{13}C of SMPD-2; (C) HSQC of SMPD-2; (D) COSY of SMPD-2; (E) HMBC of SMPD-2; (F) NOESY of SMPD-2; (G) TOCSY of SMPD-2; (H) Speculated structure of SMPD-2.

2.2 *Cytotoxic effect*

To test the potential toxicity of SMPD-2, different concentrations of SMPD-2 were incubated with macrophages and assessed the cells viability by the MTT method. As shown in., SMP was non-toxic to macrophages below 1000 μg/mL (Fig.S2 A).

2.3 *Cytokine levels*

Activated macrophages are thought to be associated with cytokines such as interferon-γ (IFN-γ) [47] and interleukin (IL-1β), and activate other cells to initiate

adaptive immune responses [48]. Compared with the PBS group, SMP-H, SMP-M, and SMP-L significantly increased the expression level of IL-1β (*P<0.01*), of which SMP-M (*P<0.001*) was the most significant (FigureS2 B). SMP-H, SMP-M, and SMP-L significantly increased the expression of INF-γ (*P<0.0001*) (Fig.S2 C). In short, three concentrations of SMP have a certain role in promoting the secretion of cytokines by macrophages.

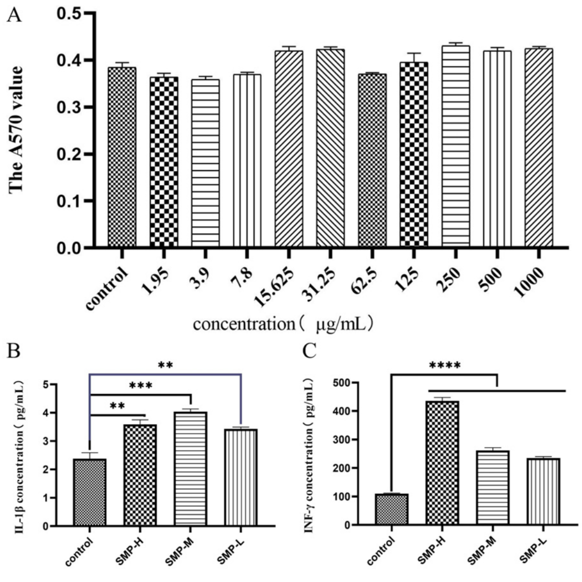

Figure S3 Cytotoxicity and cytokines of macrophages. (A) Viability of murine peritoneal macrophages after 48 h exposure to SMPD-2; (B) IL-1β, (C) IFN-γ levels were measured by ELISA. In the figure, SMP-H represents high concentration of SMPD-2, SMP-M represents medium concentration of SMPD-2, and SMP-L represents low concentration of SMPD-2. Data were expressed as the mean±SEM, *n*=4, ***p<0.01*, ****p<0.001*, *****p<0.001*.
